# A Narrative Approach to Describe QoL in Children With Chronic ITP

**DOI:** 10.3389/fped.2019.00163

**Published:** 2019-05-07

**Authors:** Paola Giordano, Giuseppe Lassandro, Nicola Antonio di Meo, Valentina Palladino, Barbara Lovrencic, Marco Spinelli, Luigi Reale, Momcilo Jankovic

**Affiliations:** ^1^Department of Biomedical Science and Human Oncology-Pediatric Unit “B. Trambusti”, University of Bari “Aldo Moro”, Bari, Italy; ^2^Italian Immune Thrombocytopenia Patients Association, Caprino Veronese, Italy; ^3^Foundation MBBM at San Gerardo Hospital, Pediatric Clinic University Milano-Bicocca, Monza, Italy; ^4^Fondazione ISTUD, Milan, Italy

**Keywords:** quality of life, parents-psychology, chronic (disease), thrombocytopenia, children

## Abstract

**Objective:** Primary immune thrombocytopenia (ITP) is a hemorrhagic disorder. Spontaneous recovery within 12 months occurs in the majority of pediatric patients. Nevertheless, in 20–30% of children the disease is chronic. The impact extends to the patients' families, whose everyday life, in terms of interpersonal relationships and financial status, is adversely affected. This study investigated the ability of a narrative instrument to improve the quality of life of pediatric chronic ITP patients and their families and quantified the familial burden imposed by the illness.

**Method:** A quantitative survey and a narrative plot delivered through an online platform were adopted for the analysis.

**Results:** Pediatricians of ten Italian Hematologic Centers explained the projects to patients and their family in the outpatient clinic. 70 caregivers of children with ITP filled the *ad-hoc* questionnaire. Data from 53 caregivers revealed the emotional impact of pediatric chronic ITP. The narrative approach highlighted the specific resources used by patients and their families to cope with the disease and its chronicity.

**Discussion:** Caregivers underlined the need for “humaneness” in their interactions with clinical personnel. The majority of respondents provided positive feedback regarding the narrative project, defining the experience as “liberating” and improving their quality of life.

## Introduction

Primary immune thrombocytopenia (ITP) is an autoimmune hematological disorder characterized by a platelet count < 100,000/μL. Recently, the ITP International Working Group consensus report, divided ITP into three phases, separating the chronic form (more than 12 months since the diagnosis) from newly diagnosed ITP (<3 months since the diagnosis) and persistent (from 3 to 12 months after the diagnosis) ITP (1). Patients with a platelet count < 30,000/μl may require treatment to prevent hemorrhages (2). The goal in patients with ITP, with or without treatment, is an acceptable clinical condition, defined as limited to very mild cutaneous hemorrhages.

Based on current estimates, the annual incidence of newly diagnosed ITP in children is between 1.9 and 6.4 per 100,000 (3). Pediatric ITP is usually of short duration, with at least two-thirds of patients recovering spontaneously within 12 months (4). However, 20–30% of children with ITP have chronic thrombocytopenia (5). Insidious onset and age at disease development > 10 years significantly influences the development of chronicity (6, 7).

The platelet count in ITP does not necessarily correlate with the bleeding risk (8), and signs and symptoms vary widely (9). One-third of the children with ITP are asymptomatic, whereas the remainder may suffer from mild or severe bleeding manifestations (10, 11).

Treatment in asymptomatic or mildly symptomatic patients consists of observation whereas patients with severe disease usually receive intravenous human immunoglobulin (IVIG) and/or steroids as a first-line treatment, and immunosuppressive agents (rituximab, mycophenolate mofetil, sirolimus) or thrombopoietin (TPO) receptor agonists (eltrombopag or romiplostim) as a second-line treatment (12, 13). Splenectomy, while effective, is a third-line therapeutic option due to the postoperative risk of infection (14).

Families of children with ITP must be on the lookout for sudden bleeding but they must also help the child cope with the side effects of treatment, as well as the related fears of specific medical procedures (frequent blood tests, invasive exams such as bone marrow aspiration and repeated intravenous or oral therapy) and perhaps even splenectomy. There may also be restrictions that compromise the family's lifestyle (15).Thus, in a child with ITP, the quality of family life and interpersonal relationships are also affected by the disease (15, 16). Moreover, the cost of caring for a child with ITP may pose an economic burden on the family (17–19). Despite international guidelines on pediatric ITP and many studies cited in literature, it is difficult to carry out comprehensive care due to limited resources and because some of the issues are directly related to the complexity of the condition (i.e., bleeding risk, available therapies).

Because health-related quality of life (HRQoL) plays a major role in the well-being of patients with chronic diseases, in this study we explored the quality of life of pediatric chronic ITP patients and their families (20, 21) without using HRQoL objective measures but delegating an answer to a narrative approach. We know that several tools have been developed to evaluate the health-related quality of life (HRQoL), quantitatively or qualitatively. The Kids' ITP Tools (KIT), developed by the Canadian researchers Robert Klaassen and Barnard et al. is a disease-specific measure of HRQoL for childhood ITP patients and their families (22, 23). It has been validated in several countries, including Italy (24), UK, France, Germany, Uruguay, Egypt, and China. However, studies based on the use of qualitative approaches to chronic childhood ITP are still lacking. On the contrary, narrative medicine is a qualitative tool in which patients' feelings about their illness are revealed through a critical analysis of their life stories (25), as recently applied in a study of leukemia patients (26).

## Objective

In this study we combined quantitative and almost qualitative approaches to investigate the quality of life of childhood ITP patients and their families. Specifically, we used narrative instruments and a quantification of the burden of the illness, including the economic costs to complete the study.

## Methods

### Patients and Study Methodology

**Figure 1:**
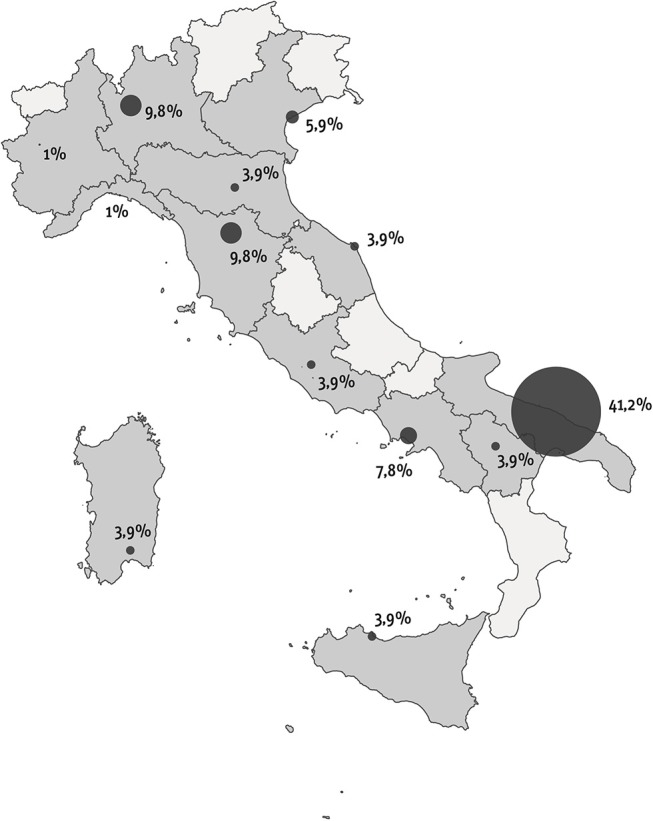
Geographical distribution of enrolled people.

This pediatric study was performed in Italy between September and December 2016 as part of a global study with two arms: one for adults with ITP and one for children with ITP. The investigative instruments used for the project were designed by a board consisting of the President of the Italian Immune Thrombocytopenia Patients Association (AIPIT onlus), the Scientific Director of the “Progetto-Ematologia” Foundation, and researchers of the Healthcare Area of “Istituto Studi Direzionali” (ISTUD) Foundation. The instruments comprised a quantitative survey and a narrative plot delivered through an online platform (Surveygizmo). Based on their expertise in the diagnosis and management of ITP and on geographic criteria to obtain a relatively homogeneous sample, 10 AIEOP Centers selected ITP patients for participation in the study (Figure 1). The project was communicated through websites, newsletters, and social media. Children affected by chronic ITP were only enrolled in sequence from each center file.

Parents on a voluntary basis (only < 10% declined invitation) provided written informed consent to participate and were then invited to access the project website (www.medicinanarrativa.eu/ITP), where they could record their experiences. To ensure comparable data, only the caregivers of children with a specific diagnosis of ITP were included in the project. An ability to communicate in Italian was indispensable for the eligibility of each participant.

### Ethical Considerations

Prior to their participation, the caregivers provided signed consent forms after being informed about the aim of the project as well as the confidential handling of both narratives and those reported form the results, as per the Italian Law on Privacy and the Safeguarding of Sensitive Data (D.Lgs n196, 2003). The project was performed in accordance with the principles of the Declaration of Helsinki.

### Data and Text Analysis

Socio-demographic variables were analyzed using descriptive statistics and included the following: (1) onset of the disease, impact of the disease on the emotional, social and economic well-being of the family; (2) clinical records, including platelet counts, treatments, and the side effects of therapy); (3) quality of life of the patient and his or her family, including emotional status, relationship to the disease, and problems at work or outside the workplace. These areas were selected after a doctors-meeting. The doctors highlighted the hard points that emerged most in the interview with parents.

Information on the burden of the illness was quantified based on the number of hours spent in caring for the patient and assessments according to the Italian National Labor Contract for careers. Depending on the level of care, the cost per hour described in the contract was 4.41€ (“level A”–generic career with basic training) or 7.83 € (“level Ds”: assistant to non-self-sufficient patient, holding a professional degree or a specific certificate). The monthly cost was set at 606.79 € for level A and 1158.42 € for level Ds, assuming a 5 h patient-related work/day.

The narratives, which were written in Italian, were investigated using the Grounded Theory approach, integrating qualitative and quantitative research, and clustered using Kleiman's methodology (27) as well as Frank's classification of texts (28, 29) Table 1 lists the questions asked.

**Table 1:** Narratives.

**YESTERDAY**
I'm talking about me…
We realized something was wrong…
When they diagnosed a thrombocytopenia, I felt …
The others seeing his body through of him/her…
**TODAY**
He/She feels today…
Move for him/her is…
Sleep for him/her is…
If I think about my son's illness I draw…
**TOMORROW**
If I had to imagine tomorrow I would like…

A quantitative analysis of the text was carried out using the NVivo software, a useful instrument for the classification of recurring words and semantic expressions in stories written in Italian (30–32). The qualitative analysis was conducted by ISTUD. Three researchers, separately, in a blinded session analyzed the answers and assigned a value for each of them. Then, ISTUD researchers compared the values and formulated a unique agreement for each of the answers. In addition, particular attention was given to “social exclusion or inclusion,” because of ethical issues. The researchers assigned corresponding metaphors describing the patients' illness, during a shared session. Each metaphor was clustered by the ISTUD researchers on the basis of the images or primary emotions represented by those words (33).

## Results

### Socio-Demographic Characteristics of ITP Patients

Pediatricians of ten Italian Hematologic Centers explained the information of the projects to patients and their families in the outpatient clinic. 17/70 questionnaires were not filled in adequately and were therefore excluded. The basic socio-demographic characteristics of the participants are summarized in Table 2. Regarding the education level of the 53 participants, the majority (71.7%) of the respondents had a university degree; the remainder had no more than a primary or secondary school education.

**Table 2:** Sociodemographic profiles.

	**Patients % (*n* = 53)**
**Number of participants**	
Men	18.9 (10)
Women	81.2 (43)
Average age	42.6 (21–54)
**Marital status**	
Unmarried	13.2 (7)
Married/living with a partner	79.2 (42)
Divorced	7.6 (4)
**Educational level**	
Primary school	3.8 (2)
Secondary school	24.5 (13)
High school	47.2 (25)
Master degree	24.5 (13)
**Working status before diagnosis**	
Employee	49 (26)
Freelance	9.4 (5)
Unemployed because of disease	1.9 (1)
Unemployed because of other causes	1.9 (1)
Housewife / household	24.5(13)
Student	13,2 (7)

### Onset of Symptoms and Diagnosis

In 96% of the patients, the platelet count was < 50,000 at the time of diagnosis.

Over 50% of the respondents reported that their child was initially asymptomatic at the time of diagnosis, such that the disease was discovered incidentally, through a blood test administered for other reasons. The remaining recalled that the child had displayed specific hemorrhaging symptoms (bruising or petechiae) consistent with those reported in the ITP literature (34–36). In 31% of the patients, fatigue was the only symptom.

### Treatment

Treatment was required by 34% of the patients. The most common therapies were IVIG (58%) and corticosteroids (27%). A few children were being treated with TPO receptor agonists (4%), other therapies (monoclonal antibodies, 8%), or other drugs (3%). Among the patients treated with IVIG, 61% were satisfied with their therapy and considered the side effects as negligible. By contrast, in the group of patients receiving corticosteroids, 43% expressed negative feelings about the treatment. The drugs administered to the patients were considered to be “extremely” (19%), “very” (35%), or “moderately” (23%) severe in term of their toxicity.

Only 5.3% of the children with ITP had undergone a splenectomy and none had surgical complications, although in one patient the surgery was ineffective.

The average follow-up frequency was 7.5 times a year, with each visit lasting an average of 35 min. In 54% of the children with ITP, the platelet count was checked once a month.

### Clusters of Narratives, Patients' Quality of Life, and Emotional Analysis

The 53 collected narratives were given by caregivers between 21 and 54 years of age and 81.1% were female.

The challenges experienced by the children in their daily activities were revealed in the survey to caregivers. Movement-related activities, sports participation, and studying were the most commonly cited aspects of everyday life that were perceived as altered/compromised (23%). The majority of the participants (62.5%) reported that their child had difficulties in studying and suffered from a lack of concentration. The narratives showed that children with chronic ITP usually give up sports or other social activities; their movement activities are subject to considerable limitation due to parental anxiety and the fear of worsening symptoms. According to 31% of the respondents, the limitations imposed by the parents were extremely relevant and had a deep impact on the child's everyday life (difficulties/limitations in movements: “he can't play football, he lost his football friends”; “Doing gymnastics at school is a problem”). Conversely, being away from home, sleeping, and swimming were not or were less impacted, according to the parents (No change in movements: “the disease didn't change his activities”; “movements are essential”; “moving for him means living”).

These data could represent a limitation because they were underlined by the parents: the need to control all the child's activities in order to prevent possible dangerous situations.

The illness metaphors evoked by the respondents in their narratives mostly conveyed negative image (threat, calm/hope, sadness, shame: “Hurricane,” “Hang in the balance,” Heart,” “Sunset,” “Darkness,” “Purplish bruises, “A stained dress”).

### Familial, Social, and Medical Relationships

One of the objectives of the social investigation of the narratives was to identify changes in the caregiver-child relationship as well as in family and social settings, such as school. Indeed, the narrations revealed social difficulties in school for 13.7% of the children as well as problems in parent-child but also in husband-wife relationships. The latter concerned the educational method, treatment modality, and disease management strategy deemed best for the child.

A deep sense of discomfort about the child's disease and people's reaction to it was described by 36% of the parents; 9% faced the problem with a great deal of pain and another 9% dealt with the situation by feeling angry at him/herself. The narrated emotions expressed by caregivers with respect to their child's relationships with others are: shame, anger, normal, sadness (“It is not nice to see a baby full of bruises”; “It is possible that there is no cure”; “What they think of me is not a problem”; “I found myself very weak, I cried after each question”).

Overall, 34% of the parents had established positive relationships with the healthcare professionals caring for their child and tended to express gratitude toward them. However, other parents expressed the need for improvements in healthcare, especially more clinical research (12%), new treatments, and fewer cortisol prescriptions, but also more empathy (22%), humanity (19%), and professional capability. Many caregivers also used the narration to suggest logistical improvements in patient management.

### Blood Count Testing and Burden of Illness

The platelets count of 54% of patients were tested approximately once a month. While the results were often communicated to the hematologist (75%), they were rarely sent to the family doctor (13%). However, the parents of 12% of the children had become experienced in interpreting the test results on their own.

The total burden of illness was the sum of the direct and indirect costs: the total costs incurred by families of ITP children were about 3000€ per year and included blood count testing, hematologic visits, drug costs, travel expenses, including transfers to reach healthcare centers, and other items (total 1,645€). Globally, the declared indirect cost to a family managing ITP was ~4645€ per patient per year, which clearly had a negative impact on the economic situation of the families.

Blood count testing requires an out of pocket payment of 10.80€ per test on average and 25.90€ per each hematologic visit. In addition, according to 48.9% of the respondents, ITP had negative consequences on the jobs or educational activities of the family.

Caregivers of ITP children were absent from work for a mean of 20 days per year, while the mean annual period in which the caregiver was on the job but produced poor-quality work was 26 days. The average time needed to reach a hematologic center (mean 68 km away) was 67 min; globally, the mean time needed for a medical visit, including going to and coming home from the center was 7.39 h. Other incurred costs were related to going to healthcare facilities (cited by 68% of participants), additional drugs (28%), and other extras (65%).

## Discussion

Taking care of children with ITP is a challenge that affects the whole family. Children with signs of an abrupt fall in the platelet count must be driven to the hospital. The average distance traveled by families to reach the referenced hematology center was 68 km, and the total time spent reaching, visiting and returning from the center was almost 8 h. These data confirm previous results regarding the impact of pediatric chronic ITP as perceived by caregivers. Those data were obtained using the KIT tool, an internationally validated quantitative instrument, in the sections addressed to parents (23, 36, 37). Our study also highlights the relational impact of the disease, as 25% of respondents reported difficulties not only in the parent-child but also in many aspects of the spousal relationship regarding the best approach to disease management and caring for the child.

The narratives of 50% of the caregivers focused on the illness, with the impact on daily life being the most prominent. Specifically, 46% of the respondents reported that the disease compromised sports participation and activity levels, and 26% reported that it complicated travel or simple play. Considering that, on average, the patients being cared for were 11 years old, an effect of ITP on childhood was to be expected and confirmed the self-reporting scores of the children who took part in previous KIT studies (23, 38, 39).

The other recurring aspect in the narratives was the emotional toll of caring for a child with ITP, as the replies were in accordance with those in the existing literature on parental perceptions of the disease. One element in previous KIT-based studies that could not be overlooked was the substantial difference between the parents vs. the children's scores regarding the emotional burden of the disease (23, 36–40). This can mainly be attributed to differences in the perceptions of happiness, as the children's scores in this regard were higher than those of their parents. Caregivers have a broader look at the concepts of serenity, happiness, health, and quality of life, and their primary concern is their children's future, including the risks that every opportunity in life will bring, regardless of the actual platelet count. Children, on the other hand, are more concerned about the restrictions on their activities than on the side effects of participating in those activities (41).

The narratives therefore revealed another limitation, one that was not directly felt by the child: the parents' need to have sufficient control over their child so as to prevent dangerous situations. In fact, 45% of parents reported feelings of fear/anxiety. This would certainly impact the child's lifestyle, by precluding participation in activities that, with minor attention, could otherwise easily be enjoyed.

Three important limitations to our study can be pointed out. First the results of the study should be considered as an “expert opinion” conducted by pediatricians qualified in ITP. Second, the narratives confirmed the emotional impact on parents related to their child's ITP, which in turn may decrease the child's HRQoL. However, the lack of a questionnaire or a narrative from the child's perspective may have resulted in an incomplete or biased perception of the child's happiness. Finally, a comparison between the two emotional experiences (parents vs. children) was not possible, in contrast to evidence-based medicine studies, which have responded to this need, as noted by Eiser and Morse (42). Second and third limitations can in part be attributed to the small sample size, which prevented sample stratification by age group. It can be assumed that the different age groups would differ in their views of quality of the life, skill learning, and relationships and development aspects. Nonetheless, even if age groups could have been established, drawing narrative information from small children is extremely difficult. Similar limitations, i.e., a low number of data entries for the part of the questionnaire completed by children, have been reported by the authors of KITs studies (39). Moreover, the majority of respondents were mothers (>80%) with a high educational level (>70% with university degree). In further studies we hope to minimize the bias and carry out a correlation with data from the affected children themselves.

Among the various forms of therapy, corticosteroids were evaluated negatively by 43% of the respondents, due to difficulties in managing the side effects, which according to 54% were very/extremely relevant. These data are consistent with those obtained through the KITs, validated in different countries (36–41), and a reminder of the limitation of considering only traditional first-line treatments (23, 39, 40). A broader outlook at the therapeutic impact would result from the interpretation of these data together with narratives describing children receiving new-generation therapies (romiplostim), by enabling comparisons of new vs. traditional drugs. This approach would be in line with current research perspectives that emphasize both the inclusion of HRQoL among the indicators used to assess the effectiveness of romiplostim (43, 44) and the need to expand the pediatric sample size to understand the potential role of this new class of drugs in chronic cases (45).

The main aim of this project was to obtain an overview of the illness burden imposed on families in Italy whose children suffer from chronic ITP. This is the first study to quantify the disease burden, with results revealing that the extra costs related to the illness may exceed 4,600€ per year. However, future studies are necessary to study the economic impact in relation to income in the population (and regional differences: Northern vs. Southern Italy). In addition, the time needed for parents to attend to their children's disease was significant. Parents also reported that feelings such as anxiety often impeded their job performance or opportunity for career advancement. In fact, several participants described serious work-related economic consequences. These data indicated a need for economic support to the families of children with ITP. Recently, the Italian Health System reformed a previous law (Gazzetta Ufficiale n. 65, 18 Marzo 2017, Art. 64) such that blood count testing is now free.

Regarding the care experience, the need for improved professional communication skills was cited, which underlines the need for greater “humanity” in the clinical world. The majority of respondents provided interesting, positive feedback on the narrative project, with many saying that they found the writing experience to be “liberating.” This well-demonstrates the potential of Narrative Medicine as an instrument that accurately reflects the patient's/caregiver's current perception of health and its centrality, in agreement with the bio-psycho-social model.

## Author Contributions

All authors listed have made a substantial, direct and intellectual contribution to the work, and approved it for publication.

### Conflict of Interest Statement

The authors declare that the research was conducted in the absence of any commercial or financial relationships that could be construed as a potential conflict of interest.

## Abbreviations

ITP: Primary Immune thrombocytopenia
HRQoL: Health-related quality of life
ISTUD: Istituto studi direzionali
KIT: Kids' ITP tool
AIEOP: Italian Association of Pediatric Hematology/Oncology
AIPIT: Italian Immune Thrombocytopenia Patients Association
TPO: Thrombopoietin
IVIG: intravenous human immunoglobulin.
