# Corrigendum: A Narrative Approach to Describe QoL in Children With Chronic ITP

**DOI:** 10.3389/fped.2019.00344

**Published:** 2019-08-26

**Authors:** Paola Giordano, Giuseppe Lassandro, Nicola Antonio di Meo, Valentina Palladino, Barbara Lovrencic, Marco Spinelli, Luigi Reale, Momcilo Jankovic

**Affiliations:** ^1^Department of Biomedical Science and Human Oncology-Pediatric Unit “B. Trambusti”, University of Bari “Aldo Moro”, Bari, Italy; ^2^Italian Immune Thrombocytopenia Patients Association, Caprino Veronese, Italy; ^3^Foundation MBBM at San Gerardo Hospital, Pediatric Clinic University Milano-Bicocca, Monza, Italy; ^4^Fondazione ISTUD, Milan, Italy

**Keywords:** quality of life, parents-psychology, chronic (disease), thrombocytopenia, children

Luigi Reale was not included as an author in the published article.

Furthermore, in the original article, there was an error. A correction has been made to **Methods, Patients and Study Methodology**, first sentence of the section. “The study was performed in Italy between September and December 2016” should read as “This pediatric study was performed in Italy between September and December 2016 as part of a global study with two arms: one for adults with ITP and one for children with ITP.”

The authors apologize for this error and state that this does not change the scientific conclusions of the article in any way. The original article has been updated.

